# Prospective functional outcomes in sequential population based cohorts of stage III/ IV oropharyngeal carcinoma patients treated with 3D conformal vs. intensity modulated radiotherapy

**DOI:** 10.1186/s40463-015-0068-4

**Published:** 2015-05-13

**Authors:** Paul Kerr, Candace L Myers, James Butler, Mohamed Alessa, Pascal Lambert, Andrew L Cooke

**Affiliations:** Department of Otolaryngology, Winnipeg, Manitoba Canada; Cancer Care Manitoba, Winnipeg, Manitoba Canada; Department of Radiology, Winnipeg, Manitoba Canada; Department of Radiation Oncology, CancerCare Manitoba, 675 McDermot Avenue, Winnipeg, Manitoba R3E 0 V9 Canada

**Keywords:** Oropharyngeal, Intensity modulated, Functional outcomes

## Abstract

**Background and purpose:**

To compare early (3 and 6 month) and later (12 and 24 month) functional outcomes of stage III and IV (M0) oropharyngeal squamous cancer patients treated in sequential cohorts with 3D conformal (3DCRT) or intensity modulated radiotherapy (IMRT).

**Patients and methods:**

200 patients in sequential population based cohorts of 83 and 117 patients treated at a single institution with 3DCRT and then IMRT respectively were prospectively assessed at pre-treatment and 3, 6, 12 and 24 months post treatment. A standard functional outcomes protocol including performance status (KPS, ECOG), 3 Performance Status scales for Head and Neck (PSS-HN), the Royal Brisbane Hospital Outcome Measure for Swallowing (RBHOMS), Voice Handicap Index-10 (VHI-10) and self-rated xerostomia were applied.

**Results:**

Mean age at diagnosis was 59 years. The primary site was base of tongue in 77 and tonsil or soft palate in 123 patients. Median follow up was 2.5 years for the second cohort. Concomitant therapy was used in 159 (79.5%). Overall survival at 3 years was 75.6% and 71.5% for IMRT and 3DCRT cohorts respectively (not significant). A multiple imputation technique was used to estimate missing values in order to avoid a healthy patient bias. KPS and ECOG reached nadirs at 3 to 6 months but approached baseline values at 12 to 24 months and did not differ by treatment. The 3 PSS-HN scales, Eating in Public (p < 0.001), Understandability of Speech (p = 0.009) and Oral Diet Texture (p = 0.002) and all showed significantly better outcomes in favor of IMRT. The RBHOMS showed a difference in favor of IMRT which appeared during 3 to 6 months (p < 0.001). The VHI-10 also showed a difference in favor of IMRT (p = 0.015). Self-rated xerostomia did not differ at 3 and 6 months but was significantly better in favor of IMRT after 12 months p = 0.005

**Conclusions:**

A prospectively administered functional outcomes protocol showed meaningful differences in favor of IMRT over 3DCRT early (3–6 months) and later (12–24 months) in the treatment of oropharyngeal carcinoma with equivalent survival. These data support the adoption of IMRT as the standard radiation treatment method for patients with stage III and IV (M0) oropharyngeal squamous carcinoma. KPS and ECOG may not be sensitive to oropharyngeal cancer patients’ functional outcomes by treatment.

## Background

Organ preservation protocols using chemo-radiotherapy for stage III and IV (M0) oropharyngeal squamous carcinoma have been developed to preserve anatomy and function. However acute and long term toxicities remain problems with the organ preservation approach. Long term toxicities related to radiation include chronic ulceration, xerostomia, pharyngeal constrictor dysfunction, esophageal stricture, impaired swallowing, PEG tube dependency, laryngeal edema and neck fibrosis [1,2]. However these specific narrow indicators of treatment related dysfunction may not necessarily correlate with broader quality of life and performance status which may be more meaningful for patients.

Intensity modulated radiation therapy [IMRT] offers an opportunity to generate dose distributions more conformal to the target volumes including tumor, involved nodes and areas at risk compared to its predecessor, 3D conformal radiotherapy (3DCRT) with relative sparing of surrounding normal tissues [3]. Reduction of the mean dose to the parotid gland by IMRT is feasible and correlates with a reduction in patient and observer-rated xerostomia [4]. Relative sparing of the pharyngeal, laryngeal and cervical esophageal swallowing structures outside the PTV is also possible with IMRT to avoid grade 3–4 late dysphagia and cervical esophageal stricture [5].

Two completed randomized phase III trials have compared the toxicity of IMRT with 3DCRT [6-8] in oropharyngeal cancer patients. The GORTEC 2004–01 is in progress [www.gortec.fr]. The trials are small, or had a heterogeneous population (i.e. included hypopharynx or larynx patients) or had endpoints that are limited to xerostomia and salivary function. One trial [8] used the EORTC QOL QLC-C30 with the Head and Neck Module (HN35) to assess 58 patients at least once. They concluded that IMRT when compared to 3DCRT resulted in clinically meaningful and statistically better QOL scores.

There are several non-randomized comparisons of IMRT vs. 2DRT or 3DCRT in head and neck patients (excluding NPC) [4,9-13] using a variety of instruments, heterogeneous populations and different time points for measurement which demonstrate patients treated with IMRT experience statistically meaningful improvements in several important QOL domains. IMRT has nevertheless been widely implemented with this limited information.

To address these issues we have prospectively collected standardized longitudinal clinical performance status and functional outcomes on 2 sequential population based cohorts of patients with stage III and IV(M0) oropharyngeal carcinoma treated at one institution.

## Methods

The methodology has been described in a previous publication regarding oropharyngeal cancer patients treated with 3DCRT at this institution to 2008 [14]. All patients diagnosed with carcinomas of the head and neck and considered for curative intent between 2003 and 2011 were reviewed in a multi-disciplinary consensus conference to confirm site, histology and TNM stage and to determine treatment intent and modality(s). Patients were included in this study if they had American Joint Committee on Cancer (AJCC) stage III or IV (M0) squamous cell carcinoma of the oropharynx and were treated with 3DCRT or IMRT (after 2007), either with or without concomitant cisplatin or cetuximab and with curative intent (typically 66–70 Gy in 2 Gy fractions to gross disease). Neck irradiation was bilateral except in highly selected patients with cancers of the tonsil with N1 disease. Over the period of the two cohorts the selection and treatment polices for oropharyngeal stage III and IV cancers did not change except for the implementation of IMRT and the addition of cetuximab in some cases not eligible for cisplatin. Neck dissection for bulky neck nodes prior to or after radiation was allowed. Patients were excluded if they were treated with surgery alone, surgery to the primary tumor (with or without post-operative radiotherapy), or had a previous head and neck cancer within five years prior to diagnosis. Human papilloma virus [HPV] infection was not assessed in the early years of the study and is not included in the analysis.

The Manitoba Cancer Registry [MCR] is a comprehensive and accredited population-based registry for 1.2 million people. The MCR is a member of the North American Association of Central Cancer Registries which administers a program that reviews member registries for their ability to produce complete, accurate, and timely data. Fields include diagnosis coded using the International Classification of Diseases 10th revision for Canada (ICD-10-CA), age, and TNM stage. Medically necessary care is freely provided to all Manitobans without premiums or co-payments and non-participation in the plan is rare [www.gov.mb.ca/health/guide/2.html]. Therefore the registry and any derived cohort can be considered complete and population-based. All incident registry cases from 2003 to 2011 inclusive with oropharyngeal cancer were reviewed to ensure all cases were included.

Patients were assessed prospectively by one Speech Language specialist (CM) using a standardized clinical functional outcomes protocol at a pre-treatment visit and post-treatment at 3, 6, 12, 24 and 36 months. In the first year, assessments could vary by as much as +/− 1 month. At 24 and 36 months visits could vary within several months. Pre-treatment assessment was not a protocol standard until March of 2005. The following data were also collected: gender, age at diagnosis, weight at each visit, date of placement and removal of feeding tube if used, chemotherapeutic agents used, amifostine use, dose of radiotherapy, date of recurrence assessed clinically, radiographically or pathologically, site of recurrence, date of death, hospitalizations, tobacco use, and incidence of respiratory infections.

### Functional outcomes protocol

The protocol was intended to capture functional outcomes beyond recurrence and survival consistent with the International Classification of Functioning, Disability and Health (ICF) 22–23. The protocol included 8 instruments which have proven inter-rater reliability and validity and are widely used in head and neck cancer patient assessments. The protocol was applied prospectively, in face to face interviews with patients by a single speech language pathologist (CM).

The Karnofsky Performance Status (KPS) scale [15] and the Eastern Cooperative Oncology Group (ECOG) toxicity and response criteria scale [16] are clinician-rated standard tools used to assess performance in activities of daily living.

The Performance Status Scale for Head and Neck Cancer Patients (PSS-HN) [17] is a clinician rated interview assessment tool that describes performance on three subscales: Eating in Public, Understandability of Speech and maximum Oral Diet Texture. Each PSS-HN scale has a 5 or 10 point ordinal scale (nominally from 0 to 100) with higher scores indicating better performance. The PSS-HN has good inter-rater reliability and ability to discriminate levels of functioning [18]. For graphic purposes but not for analysis the PSS-HN ordinal scales are here represented as 4 ranges for Eating in Public PSS-HN and Oral Diet Texture PSS-HN and 3 ranges for Understandability of Speech PSS-HN.

The Royal Brisbane Hospital Outcome Measure for Swallowing (RBHOMS) [19] is a clinician-rated 10-point scale which measures oral intake, swallowing function and relative dependence on enteral tube feeding. For graphic purposes the scale was collapsed into 4 ranges. The ranges were 1–3, (total tube dependence, NPO), 4–5, (reduced oral intake requiring partial or total tube feed supplementation), 6–7, (modified diet with no tube supplementation), and >8, (oral intake at optimum level).

The Voice Handicap Index-10 (VHI-10) [20] is a patient-rated scale with 10 questions with answers ranging from 0 (never) to 4 (always) for a total of 40 possible points with a higher score indicating worse self-perceived voice handicap. The VHI-10 has been collapsed into 3 ranges, 0–9 (never to almost never severe), 10–19 (sometimes severe), and > 20 (more than sometimes severe).

Finally self-rated xerostomia on a 0–10 scale from the Edmonton Self-Assessment Scale (ESAS) [21] was collected.

### Statistical analysis

Overall survival (OS) and disease-free survival (DFS), for which events are recurrence without death and death from any cause, were measured from the time of diagnosis and compared by the logrank test. Patients with recurrence were followed and assessed until death or end of study follow-up. Patients were censored at the time of death, with or without recurrence.

Times for functional outcome assessments were measured from the date of the last radiation treatment. Compliance with the functional outcome protocol was measured as the percentage of patients administered a questionnaire out of all patients alive at that time. Missing assessments while patients were alive were considered to be missing at random (MAR) if they were the result of a conflict in schedule or because assessments were not systematically scheduled for pretreatment, 3 and 6 months at the beginning of data collection. Missing values were estimated using a multiple imputation bootstrap method [22]. Because the outcomes were skewed, the ICE procedure in *Stata 11.2* (StataCorp LP, College Station Tx) [23] was used for multiple imputations, which uses a conditional density approach. Pre-treatment outcomes were imputed first, using gender, stage, subsite, and age as predictors. Based on Graham [24] 20 imputations were used.

Post-treatment outcomes were then merged to the new pre-treatment dataset and imputed once, using gender, stage, subsite, age, month of assessment, and pre-treatment assessment as predictors. Results by treatment were compared with mixed logistic or ordinal models using GLLAMM in *Stata*. Adjustments to comparisons by treatment were made for variations in pretreatment function.

Xerostomia was analyzed by mixed quantile regression using lqmm in **R** 2.15.2.

This study was approved by the University of Manitoba Health Research Ethics Board.

## Results

From 2003 to 2011, 200 patients with stages III and IV (M0) squamous cell carcinoma of the oropharynx were treated with curative intent using 3DCRT (83) or IMRT (117) with or without concomitant therapy. Patient and treatment characteristics are shown in Table 1. One patient had a suspected tonsil primary but had only CIS on biopsy but had grossly involved regional nodes and has been included. Two significant differences were noted between the cohorts. Cetuximab was introduced for some IMRT patients not eligible for cisplatin (typically older or with renal impairment) and the lower number of 3 and 6 month assessments done in the 3DCRT group.

**Table 1:** **Sequential cohort patient characteristics**

		**IMRT**	**3DCRT**	
		**(N = 117)**	**(N = 83)**	***p***
Age	mean (SD)	59.9 (9.0)	58.8 (9.5)	0.41
Gender	F (%)	18 (15.4)	19 (22.9)	0.20
	M	99 (84.6)	64 (77.1)	
T stage	1 or 0* (%)	28 (23.9)	14 (16.9)	0.52
	2	42 (35.9)	30 (36.1)	
	3	28 (23.9)	20 (24.1)	
	4	19 (16.2)	19 (22.9)	
N Stage	0 (%)	15 (12.8)	12 (14.5)	0.28
	1	16 (13.6)	18 (21.7)	
	2	83 (70.9)	51 (61.4)	
	3	3 (2.6)	2 (2.4)	
Stage	III (%)	20 (17.1)	19 (22.9)	0.37
	IV	97 (82.9)	64 (77.1)	
Primary site	Tongue Base (%)	41 (35.0)	36 (43.4)	0.24
	Tonsil, soft palate	76 (65.0)	47 (56.6)	
Concomitant	Cisplatin (%)	85 (72.6)	62 (74.7)	0.01
	Cetuximab	12 (10.3)	0 (0)	
	No	20 (17.1)	21 (25.3)	
Assessments	3 months (%)	95.7	72	<0.0001
	6	98.2	90.2	0.02
	12	92.2	90.8	0.96
	24	91.2	95.3	0.49

*1 patient had grossly involved tonsil but bx showed only CIS with gross nodes.

**Figure 1:**
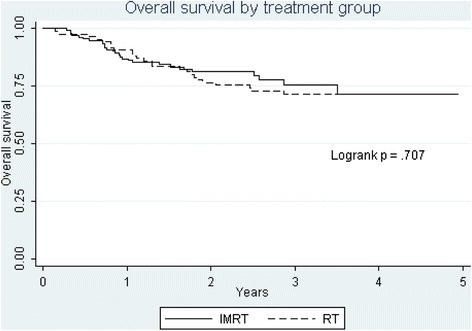
Overall survival.

**Figure 2:**
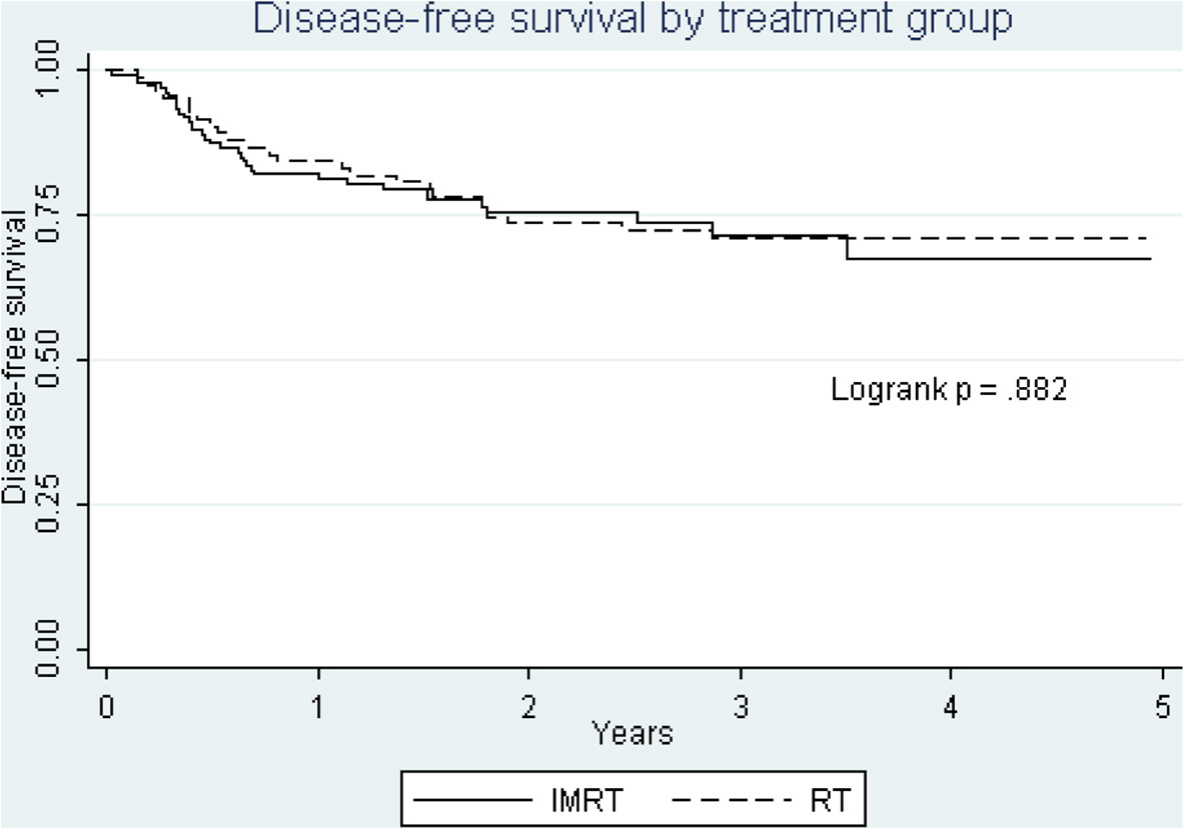
Disease free survival.

Median follow up is approximately 2.5 and 3.5 years for IMRT and 3DCRT patients respectively. Overall survival (OS) and disease-free survival (DFS) by treatment are shown in Figures 1 and 2. There was no significant difference between IMRT and 3DCRT for either OS (76 vs. 71% *p* = 0.71) or DFS (72 vs. 71% *p* = 0.88 ). Cause specific mortality (not shown) at 3 years was also not significantly different (17 vs 21%). Recurrence of any sort carried a poor prognosis. Only 1 patient with a local recurrence and 1 patient with a regional recurrence had successful surgical salvage and were alive without disease at the last follow up.

### Functional outcomes

**Figure 3:**
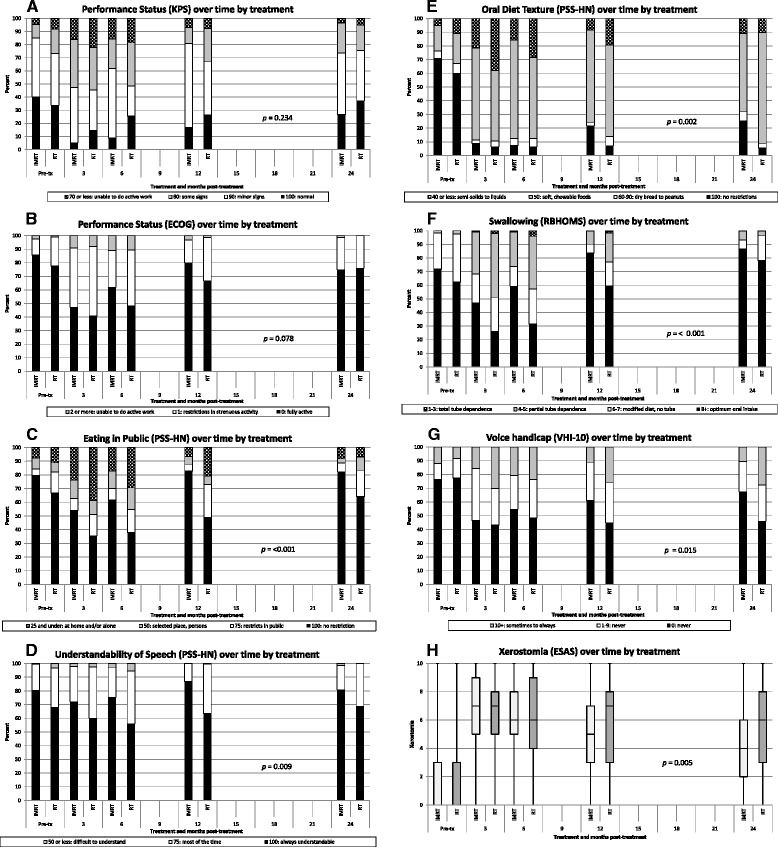
Functional outcomes in sequential population based cohorts by treatment. **A**. Performance status (KPS) over time by treatment. **B**. Performance status (ECOG) over time by treatment. **C**. Eating in public (PSS-HN) over time by treatment. **D**. Understandability of Speech (PSS-HN) over time by treatment. **E**. Oral diet texture (PSS-HN) over time by treatment. **F**. Swallowing (RBHOMS) over time by treatment. **G**. Voice handicap (VHI-10) over time by treatment. **H**. Xerostomia (ESAS) over time by treatment.

Functional outcomes are shown to 2 years of follow up. KPS is shown in Figure 3A. There is a nadir at the 3 month assessment with recovery to approximately pre-treatment values by 24 months but there is no difference by treatment (*p* = 0.234). ECOG is shown in Figure 3B. Similarly there is a nadir with recovery but there is only a non-significant trend by treatment in favor if IMRT (*p* = 0.078). On the other hand (data not shown) both KPS and ECOG were significantly predictive of death from any cause within 1 year irrespective of treatment.

All the following comparisons have been adjusted for baseline differences and death. PSS-HN results are shown in Figures 3C,D and E. Eating in Public PSS-HN (Figure 3C) shows a significant difference in favor of IMRT predicting no restriction vs. any restriction (100 vs. 75 and lower) OR 0.164 (95 %CI, 0.07-0.39 *p* = <0.001). For Eating in Public PSS-HN, at 12 and 24 months 83% and 82% of IMRT survivors had no restrictions compared to 49% and 64% of 3DCRT survivors respectively. The Understandability of Speech PSS-HN (Figure 3D) shows only <4% of patients with speech that was difficult to understand pretreatment and in the first 6 months. Thereafter no survivor was difficult to understand. Nevertheless a significant benefit in favor of IMRT (100 vs. 75 and lower) was noted, OR 0.294 (95% CI, 0.12-0.73 *p* = 0.009). Oral Diet Texture PSS-HN (Figure 3E) shows marked deterioration in both groups that did not recover to baseline even at 24 months with large subgroups preferring soft chewable food regardless of treatment. While little difference by treatment is seen at 3 and 6 months, there is a difference in favor of IMRT appearing at 12 and 24 months with an overall significant difference in favor of IMRT, OR 0.346 (95% CI, 0.18-0.67 *p* = 0.002). At 12 and 24 months 22% and 25% in the IMRT group had no Oral Diet Texture restrictions compared to 7% and 5% for 3DCRT survivors respectively.

The RBHOMS (Figure 3F) shows that pretreatment, fewer than 3% of patients in either group had partial or complete tube dependency. At 3 months, 46% in the 3DCRT group and 21 % in the IMRT group were partially or completely tube dependent. By 24 months partial tube dependency had declined to 3% in the 3DCRT group and 5% in the IMRT group and no survivor was totally tube dependent. Overall there was a significant benefit in favor of IMRT (8 vs. 7 or lower) OR 0.138 (95% CI, 0.06-0.33 *p* < 0.001).

The VHI-10 (Figure 3G) shows a significant benefit in favor of IMRT (0, 1–9 and 10+) OR 0.492 (95% CI, 0.28-0.87 *p* = 0.015) but function did not return to pretreatment levels.

Self-reported xerostomia (Figure 3H) shows little no difference by treatment at the 3 and 6 month assessments but there is a significant time-treatment interaction with improvements in favor of IMRT appearing at 12 months onward with a difference in median score of −1.232 (95% CI, −2.09—0.37 *p* = 0.005)

## Discussion

The widespread implementation of IMRT in place of 3DCRT for head and neck cancer has been justified by surrogate end points such as dose volume histograms and uncontrolled series with various endpoints. Limited randomized controlled information comparing 3DCRT to IMRT is available and only some of that data pertains to oropharyngeal carcinoma. Because narrow measures of salivary function or swallowing may not necessarily reflect patients’ perceptions or overall functional outcome acutely or longer term, we have applied a functional outcomes protocol in order to more broadly assess outcomes. The population-based nature of our data implies that case selection beyond the actual indications for treatment has not occurred and that these results may be reliable and generalizable. Further, the prospective interview and self-rating based data are additional features that suggest our data are reliable indicators of patient function at various times after treatment.

One weakness of the study is that compliance was not 100% for all assessments and lower at 3 and 6 months for 3DCRT (Table 1). We have attempted to address this possible shortcoming by a multiple imputation technique that takes into account individual patient’s scores to estimate missing values. At 12 and 24 months, where long term toxicities become important, assessment compliance was equivalent. Another weakness of the study is that for graphic purposes (but not for the statistical model analysis) we have censored patients who have died between assessments. Because death also censors functional assessments, some of the functional “improvement” seen over the months of follow up is due not only to recovery from the effects of treatment in survivors, but will instead be due to the deaths of those who are likely to have poor function. Thus the changes in functions graphically represented over time after treatment cannot be looked at as simply recovery of the cohort, but only a statement of the function of the survivors at certain specific times after treatment. Also, the two cohorts differed somewhat in their baseline pre-treatment scores. As much as possible, we have adjusted the comparisons for the differences in the pre-treatment scores and the OR and *p* values we report are adjusted. We did not report on HPV because in the early years of the study this was not routinely measured. The similar demographics and survival of the two cohorts (Table 1, Figures 1 and 2) suggest that HPV status which can profoundly affect outcome [25] did not differ greatly between the two groups. Finally cetuximab was introduced during the IMRT cohort, primarily for those who would not be able to take cisplatin, such that percentage receiving cisplatin remained much the same in both cohorts, 74.7 vs 72.6%. If cetuximab added toxicity this would bias the results against IMRT.

We have documented both early post-treatment functional impairments (3 and 6 months) and those that occur later (12 and 24 months). The RBHOMS shows significant differences that appear soon after treatment at 3 to 6 months and the Eating in Public PSS-HN, Oral Diet Texture PSS-HN and self-rated xerostomia scale show differences that mostly appear at 12 to 24 months. IMRT in our study is therefore broadly superior to 3DCRT by different measures at different times and provides a persistent meaningful benefit to patients.

The effect of treatment on these functional outcomes is likely due to the effect of (chemo) radiation on multiple end points in composite including oropharyngeal mucous membranes, pharyngeal constrictors, taste and salivary gland function [7,26] but we cannot ascertain the degree to which any of these or any other component of eating contributes to these functional endpoints. Presumably the additional conformality afforded by IMRT in comparison to 3DCRT is responsible for the differences seen in our study.

## Conclusions

In sequential population-based cohorts of 200 stages III and IV (M0) oropharyngeal cancer patients treated with 3DCRT and IMRT, with or without concomitant therapy OS and DFS did not significantly differ by treatment. While both treatments resulted in functional impairment at 3–6 months after treatment, significant differences were seen at 3 and 6 months in favor of IMRT as measured by RBHOMS, and later at 12 and 24 months as measured by the PSS-HN scales for Eating in Public, Oral Diet Texture, Understandability of Speech, the Voice Handicap Index (VHI) and xerostomia as assessed by the Edmonton Self-Assessment Score (ESAS). KPS and ECOG however did not show any differences by treatment. However, KPS and ECOG may not be sensitive to oropharyngeal cancer patients’ functional outcomes by treatment.

IMRT maintained efficacy of treatment with improved functional outcomes indicating an improved therapeutic ratio compared to 3DCRT in patients with oropharyngeal squamous carcinoma stage III and IV(M0). These data support IMRT as the standard of care for curative (chemo) radiation for such patients.

It is unlikely given the established place IMRT has in the treatment of head and neck cancer that much additional data will be available from randomized controlled trials of IMRT vs. 3DCRT apart from the pending GORTEC 2004–01 trial.
